# Motor Phenotype of Decline in Cognitive Performance among Community-Dwellers without Dementia: Population-Based Study and Meta-Analysis

**DOI:** 10.1371/journal.pone.0099318

**Published:** 2014-06-09

**Authors:** Olivier Beauchet, Gilles Allali, Manuel Montero-Odasso, Ervin Sejdić, Bruno Fantino, Cédric Annweiler

**Affiliations:** 1 Division of Geriatric Medicine, Department of Neuroscience, Angers University Hospital, Angers, France; 2 Department of Neurology, Geneva University Hospital and University of Geneva, Geneva, Switzerland; 3 Gait and Brain Lab, Parkwood Hospital, Lawson Health Research Institute, London, Ontario, Canada; 4 Department of Electrical and Computer Engineering, Swanson School of Engineering, University of Pittsburgh, Pittsburgh, Pennsylvania, United States of America; 5 Robarts Research Institute, Department of Medical Biophysics, Schulich School of Medicine and Dentistry, the University of Western Ontario, London, Ontario, Canada; University Of São Paulo, Brazil

## Abstract

**Background:**

Decline in cognitive performance is associated with gait deterioration. Our objectives were: 1) to determine, from an original study in older community-dwellers without diagnosis of dementia, which gait parameters, among slower gait speed, higher stride time variability (STV) and Timed Up & Go test (TUG) delta time, were most strongly associated with lower performance in two cognitive domains (i.e., episodic memory and executive function); and 2) to quantitatively synthesize, with a systematic review and meta-analysis, the association between gait performance and cognitive decline (i.e., mild cognitive impairment (MCI) and dementia).

**Methods:**

Based on a cross-sectional design, 934 older community-dwellers without dementia (mean±standard deviation, 70.3±4.9years; 52.1% female) were recruited. A score at 5 on the Short Mini-Mental State Examination defined low episodic memory performance. Low executive performance was defined by clock-drawing test errors. STV and gait speed were measured using GAITRite system. TUG delta time was calculated as the difference between the times needed to perform and to imagine the TUG. Then, a systematic Medline search was conducted in November 2013 using the Medical Subject Heading terms “Delirium,” “Dementia,” “Amnestic,” “Cognitive disorders” combined with “Gait” OR “Gait disorders, Neurologic” and “Variability.”

**Findings:**

A total of 294 (31.5%) participants presented decline in cognitive performance. Higher STV, higher TUG delta time, and slower gait speed were associated with decline in episodic memory and executive performances (all P-values <0.001). The highest magnitude of association was found for higher STV (effect size  =  −0.74 [95% Confidence Interval (CI): −1.05;−0.43], among participants combining of decline in episodic memory and in executive performances). Meta-analysis underscored that higher STV represented a gait biomarker in patients with MCI (effect size  =  0.48 [95% CI: 0.30;0.65]) and dementia (effect size  = 1.06 [95% CI: 0.40;1.72]).

**Conclusion:**

Higher STV appears to be a motor phenotype of cognitive decline.

## Introduction

There is growing evidence that decline in cognitive performance results in gait deterioration [1]–[3]. Commonly described in later stages of dementia, lower gait performance may be also detected early in the progression of dementia and even before the prodromal stage of mild cognitive impairment (MCI) [1], [2], [4]–[9]. This suggests that there is a motor phenotype of decline in cognitive performance, which could be used to improve the prediction of dementia.

Two main gait parameters have been related to the severity of the decline in cognitive performance (i.e., from cognitively healthy individuals [CHI] to patients with dementia): gait speed and stride-to-stride variability of stride time (STV) [1], [2], [4], [10], [11]. Slower gait speed is observed among people with dementia compared to those without dementia, and it may predict the onset of dementia [4], [10], [11]. Verghese et al. recently described the “Motoric Cognitive Risk (MCR)” syndrome, which combines a cognitive complaint and a slow gait speed [4]. The authors showed in a large sample of healthy older community-dwellers that this syndrome identified the individuals at high risk of dementia - especially vascular dementia [4]. The second gait parameter that is likely related to cognition is higher gait variability, and in particular STV, which is a measure of the reliability of lower-limb movements, is also likely related to cognition [2], [12]. For instance, higher STV has been associated with diminished executive function among CHI, and higher STV appears to be indicative of patients with MCI or mild Alzheimer's disease (AD) [5]–[7], [13], [14].

In parallel to the quantitative measure of gait parameters, motor imagery, which is defined as mentally simulating a given action without actual execution, is an accurate reflection of the higher-level control of gait [15]–[17]. For instance, using the Timed Up & Go test (TUG), it has been observed that older adults with cognitive decline imagined the TUG much faster than they actually performed it, as illustrated by an increased TUG delta time (i.e., the time difference between performing and imagining the TUG) [17], [18].

These three gait biomarkers (slower gait speed, higher STV, and impaired motor imagery of gait) are common in the course of cognitive decline. It is still unknown which gait biomarker is associated with which cognitive domain. As gait variability has been previously associated with cortical metabolic functioning in patients with MCI [19], we hypothesized that higher STV could serve as a gait biomarker of decline in cognitive performance among individuals without the diagnosis of dementia. Our objectives were: 1) to determine, from an original study in older community-dwellers without the diagnosis of dementia, which gait parameter, among slower gait speed, higher STV, and TUG delta time, were most strongly associated to lower performance in two cognitive domains (i.e., episodic memory and executive function); and 2) to quantitatively synthesize, using a systematic review and meta-analysis, the association between gait performance and cognitive decline (i.e., MCI and dementia).

## Methods

### Original study

#### Population and study design

Between January 2008 and April 2012, 4192 community-dwellers were recruited in the French Health Examination Center (HEC) in Lyon, France. From the 4192 participants, 934 (22.3%) individuals without dementia performed a quantitative gait assessment using the GaitRite system and were included in this study using a cross-sectional design. Participants were excluded if they had a history of dementia, used anti-dementia drugs or had a score ≤4 on the Short version of the Mini-Mental State examination (SMMSE) [20]. The other exclusion criteria were institutionalization, inability to understand and speak French, acute medical illness during the past month, missing clinical examination, neurological diseases including Parkinson's disease, cerebellar disease, myelopathy, peripheral neuropathy, and major orthopaedic diagnoses (e.g., osteoarthritis) involving the lumber vertebra, pelvis or lower extremities, inability to walk 6 meters unassisted and being younger than 65 years of age.

#### Clinical Assessment

Baseline assessments included a full medical examination along with collecting age, gender, and measures of height and weight. Body mass index (BMI, in kg/m^2^) was calculated based on anthropometry measurements (i.e., weight in kilograms and height in meters). The number of drugs taken daily and the use of psychoactive drugs including benzodiazepines, antidepressants, or neuroleptics, were also recorded. Lower limb proprioception was evaluated with a graduated tuning fork placed on the tibial tuberosity [21]. The mean value obtained for the left and right sides was used in the present data analysis. Distance binocular vision was measured at 5 m with a standard Monoyer letter chart [22]. Vision was assessed with corrective lenses on if needed. Depression was evaluated with the use of the 4-item Geriatric Depression Scale (GDS) score [23]. A score ≥1 indicated the presence of depressive symptoms. Episodic memory was assessed using the short version of SMMSE with scores ranged from 0 (i.e., worst performance) to 6 (i.e., best performance) [20]. A SMMSE score of 5 was used to designate decline in episodic memory performance. Executive function was assessed using the clock-drawing test, and low executive performance was considered if one or more errors were made in the execution of drawing the face of the clock and/or the hands of the clock [24]. STV and gait speed were measured at steady state walking using GAITRite-system (GAITRite Gold, CIR Systems, PA, USA) in a 6-meter corridor. The GAITRite-System is an electronic walkway-integrated and pressure-sensitive electronic surface of 5.6×0.89 m that is connected to a personal portable computer via an interface cable. Participants walked one trial at their usual self-selected walking speed in a quiet, well-lit environment wearing their own footwear according to European guidelines for spatio-temporal gait analysis in older adults [25]. Before the assessment in HEC, all participants were contacted by mail and informed not to wear high-heel shoes. Coefficient of variation (CoV  =  (standard deviation/mean) × 100) of stride time and mean value of gait speed were used as outcomes. Furthermore, participants were asked to perform the TUG at their self-selected normal speed in a well-lit environment. They all completed one trial for the TUG and then followed by the imagery of TUG: performing the TUG, then imagining the TUG while sitting in a chair. The times for each condition were recorded with a stopwatch to the nearest 0.01 second. Before testing, a trained evaluator gave standardized verbal instructions regarding the test procedure. Participants were seated, allowed to use the armrests to stand up and instructed to walk three meters, turn around, walk back to the chair and sit down. The stopwatch was started on the command “ready-set-go” and stopped as the subject sat down. For the imagined condition, participants sat in the chair and were instructed to imagine performing the TUG (iTUG) and to say “stop” out loud when they were finished. Participants could choose to do the iTUG with their eyes opened or closed, and they were not instructed on the modality of mental imagery.

**Standard Protocol Approvals, Registrations, and Patient Consents.** Participants in the study were included after having given their written informed consent for research. The study was conducted in accordance with the ethical standards set forth in the Helsinki Declaration (1983). The entire study protocol was approved by Lyon Sud-Est III local Ethical Committee, France.

#### Statistical analysis

The participants' characteristics were summarized using means and standard deviations or frequencies and percentages, as appropriate. Normality of data distribution was checked using a skewness-kurtosis test. As the number of observations was > 40 for each group, no transformations were applied to the variables of interest. For the current analysis, participants were classified into 4 groups, as follows: CHI, individuals with low episodic memory performance, individuals with low executive performance, and individuals with both low episodic memory and executive performance. First, between-group comparisons were performed using one-way analysis of variance (ANOVA) with Bonferroni corrections or Chi-square test, as appropriate. Second, univariate and multiple logistic regression analyses were performed to examine the association between each cognitive impairment (i.e., memory, or executive, or memory plus executive) (dependent variables) and each gait parameter (i.e., gait speed, STV and TUG delta time) (independent variables) adjusted on participants' characteristics (i.e., age, gender, number of drugs used per day, use of psychoactive drugs, depression symptoms, BMI, lower-limb proprioception, distance vision score, and handgrip strength). Third, we graphed the “effect size” of the difference between gait parameters (i.e., gait speed, STV, and delta TUG) in participants with low cognitive performance and those without (Review Manager version 5.1, The Nordic Cochrane Centre, Copenhagen, Denmark). P-values less than 0.05 were considered as statistically significant. All statistics were performed using SPSS (version 19.0; SPSS, Inc., Chicago, IL).

### Systematic Literature search and meta-analysis

#### Literature search

An English and French systematic Medline search limited to humans was conducted in November 2013 using the Medical Subject Heading (MeSH) terms “Delirium”, “Dementia”, “Amnestic”, “Cognitive disorders” combined with “Gait” OR “Gait disorders, Neurologic” and “Variability”. An iterative process was used to ensure all relevant articles were obtained. A further hand search of bibliographic references of considered papers and existing reviews was also conducted to identify potential studies not captured in the electronic database searches.

#### Study selection

**Figure 1:**
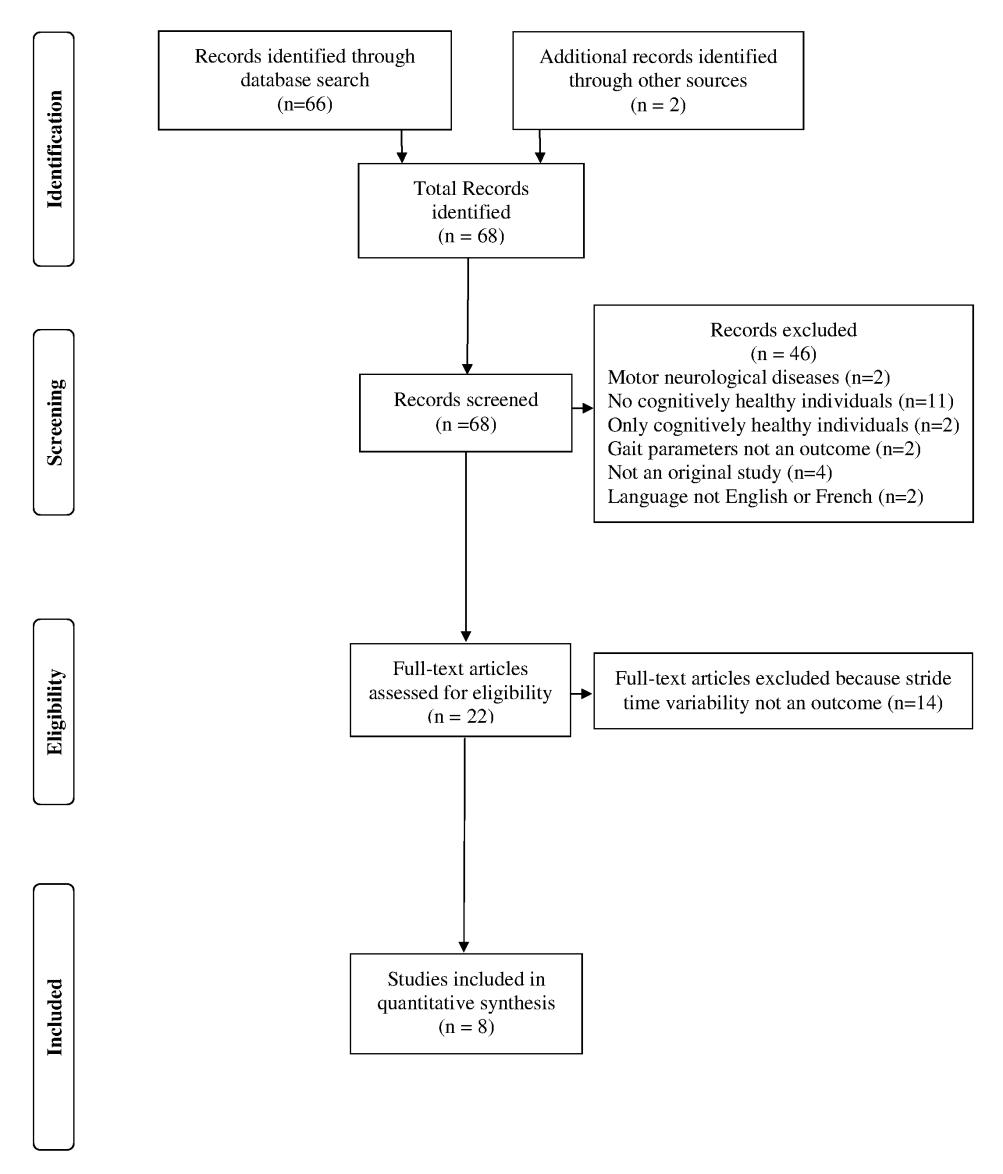
Flow diagram of selection process for selected studies included in the meta-analysis.

One member of the team (Olivier Beauchet) screened abstracts from the initial search and obtained records deemed potentially relevant. Initial screening criteria for the abstracts were: 1) observation studies (case report, case series, cross-sectional, case-control, and cohort studies were included), 2) intervention studies, 3) data collection of gait and cognition, 4) two groups of participants including cognitively healthy individuals and individuals with MCI or dementia, and 5) articles in English and French. If a study met the initial selection criteria or its eligibility could not be determined from the title and abstract (or abstract not provided), the full text was retrieved. Two reviewers (Olivier Beauchet and Cédric Annweiler) then independently assessed the full text for inclusion status. Disagreements were resolved by a third reviewer (Gilles Allali). The full articles were screened using the STrengthening the Reporting of OBservational studies in Epidemiology (STROBE) checklist, which describes items that should be included in reports of cohort studies [26] and the Consolidated Standards of Reporting Trials (CONSORT) statement for clinical trials [27]. Final selection criteria were therefore applied when STV (i.e., the gait parameter identified as the most strongly associated with lower cognitive performance in the original study) and cognitive performance were measured. The study selection is shown in a flow diagram (Figure 1).

#### Qualitative analysis

Of the 68 originally identified abstracts, 46 (67.7%) studies were not retained because 25 studies focused on motor neurological diseases (i.e., Parkinson's disease, Huntington's disease or idiopathic normal pressure hydrocephalus); there was no CHI as control group in 11 studies; there was no participants with cognitive decline in 2 studies; spatio-temporal gait parameters were not outcomes in 2 studies; 4 studies were not original studies; and 2 studies were written in a language other than English or French. Thorough examination of the 22 studies that met the initial inclusion criteria, 14 studies (63.6%) were excluded because STV was not an outcome. The remaining 8 studies were included in the systematic review [5], [7], [28]–[33].

#### Meta-analysis

All studies addressing CoV of stride time in CHI and in individuals with cognitive decline were meta-analyzed. We distinguished groups with MCI and with dementia, as these are two distinct clinical entities that differ, among others, on the level of functionality. When applicable, we also distinguished participants with amnestic MCI and participants with non-amnestic MCI [33], as well as demented patients with executive dysfunction and demented patients without executive dysfunction [32]. All results were expressed in terms of a bias corrected “effect size” of the difference between gait parameters in cases with MCI or dementia and cognitively healthy controls. An effect size calculator worksheet was used to derive effect sizes from mean, standard deviation, and size of each group (Coe's Calculator retrieved November 25, 2013 from http://www.cemcentre.org/evidence-based-education/effect-size-calculator). Qualitative descriptors of the obtained effect sizes were: less than 0.3, small; 0.4 to 0.8, moderate, and greater than 0.8, large [34]. Fixed and random effects meta-analyses were performed on the estimates to generate summary values (Review Manager version 5.1, The Nordic Cochrane Centre, Copenhagen, Denmark). Results are presented as a forest plot. Heterogeneity between studies was assessed using Cochran's Chi-squared test for homogeneity (Chi^2^), and amount of variation due to heterogeneity was estimated by calculating the I^2^ [35].

## Findings

A total of 294 (31.5%) participants presented decline in cognitive performance. One hundred fourteen (12.2%) had low episodic memory performance, 136 (14.6%) had low executive performance and 44 (4.7%) had low performance in these two cognitive domains. Participants with low cognitive performance were older than those with normal cognitive performance (P<0.03) (Table 1). Participants with low executive performance took more drugs and had a higher BMI than those with normal cognitive performance (P = 0.011 and P = 0.005). Those with low episodic memory performance used more frequently psychoactive drugs compared to those with normal cognitive performance (P<0.001). Participants with low performance either in executive function or in executive function plus memory had more frequently depressive symptoms (P = 0.035 and P = 0.002), and had also a lower distance vision (P<0.001 and P = 0.0024), lower handgrip strength (P = 0.024 and P = 0.033), lower gait speed (P<0.001) but a higher TUG delta time (P<0.001 and P = 0.016) compared to CHI. In addition, participants combining low performance in episodic memory and executive function had specifically more frequently worse lower-limb proprioception (P = 0.020) than CHI. Compared to participants with low performance in executive function, those with low memory performance had a higher distance vision score (P = 0.024) and lower TUG delta time (P<0.001). Participants with a low episodic memory performance had a higher distance vision score compared to those combining low performances in episodic memory and executive function (P<0.001). In final, STV was higher in participants with a low cognitive performance, whatever the cognitive domain, compared to those with a normal cognitive performance (P<0.003). In addition, those cumulating low episodic memory and executive performance had a higher STV compared to those with a low executive performance (P = 0.010).

**Table 1:** Comparisons of participants separated according to their cognitive performance (n = 934).

Characteristics	Total population (n = 934)	Decline in cognitive performance				P-value ||						
		No (n = 640)	Episodic memory* (n = 114)	Executive function† (n = 136)	Episodic memory & executive function‡ (n = 44)	All	No decline in cognitive performance versus decline in			Episodic memory* versus Executive function†	Episodic memory* verus Episodic memory & executive function‡	Executive function† verus Episodic memory & executive function‡
							Episodic memory*	Executive function†	Episodic memory & executive function‡			
Age (years), mean ± SD	70.3±4.9	69.8±4.4	71.2±5.4	71.6±5.7	71.6±6.0	**<0.001**	**0.005**	**<0.001**	**0.020**	0.576	0.963	0.656
Female gender, n (%)	487 (52.1)	331 (51.7)	64 (55.7)	72 (52.9)	20 (45.5)	0.697	-	-	-	-	-	-
Number of drugs per day, mean ± SD	2.8±2.4	2.7±2.4	3.1±2.5	3.3±2.6	3.2±2.4	**0.036**	0.126	**0.011**	0.208	0.503	0.818	0.800
Use of psychoactive drugs ¶, n (%)	160 (17.1)	90 (14.1)	32 (27.8)	28 (20.6)	10 (22.7)	**0.001**	**<0.001**	0.054	0.116	0.180	0.514	0.762
Depression symptoms #, n (%)	224 (24.0)	135 (21.1)	135 (27.0)	40 (29.4)	18 (40.9)	**0.006**	0.162	**0.035**	**0.002**	0.667	0.088	0.156
Body mass index, mean ± SD (kg/m^2^)	26.2±4.0	26.0±3.9	26.2±3.8	27.1±4.2	26.1±4.5	**0.048**	0.664	**0.005**	0.889	0.081	0.900	0.161
Lower-limb proprioception score § (/8), mean ± SD	6.5±1.8	6.5±1.8	6.4±1.7	6.5±1.7	5.9±2.0	0.138	0.600	0.854	**0.020**	0.777	0.081	**0.047**
Distance vision acuity ** (/10), mean ± SD	6.9±2.1	7.0±2.1	7.1±2.0	6.5±2.2	5.5±2.2	**<0.001**	0.531	**0.018**	**<0.001**	**0.024**	**<0.001**	**0.006**
Handgrip strength †† (N.m^-2^), mean ± SD	31.0±10.5	31.6±10.7	30.6±10.8	29.4±9.2	28.1±9.3	**0.031**	0.360	**0.024**	**0.033**	0.345	0.177	0.490
Motor biomarker												
Walking speed ‡‡(cm/s), mean ± SD	109.6±22.7	111.9±20.8	107.5±25.9	104.3±23.9	98.2±29.7	**<0.001**	0.053	**<0.001**	**<0.001**	0.261	**0.019**	0.115
Stride time variability ¶¶ (%) mean ± SD	2.3±2.7	2.0±1.8	3.0±3.5	2.6±3.0	3.8±6.7	**<0.001**	**0.001**	**0.025**	**<0.001**	0.271	0.080	**0.010**
Delta time of timed up & go ## (%), mean ± SD	32.4±32.8	29.3±31.3	30.9±29.7	45.4±37.6	41.5±36.2	**<0.001**	0.619	**<0.001**	**0.016**	**<0.001**	0.066	0.487

*: abnormal score on the Short Mini-Mental State Examination (score  =  5); †: abnormal score on the clock drawing test; ‡: participants with abnormal score on the Short Mini-Mental State Examination and the clock drawing test; ||: based on analysis of variance with LSD correction or Chi-square test, as appropriate; ¶: Use of benzodiazepines or antidepressants or neuroleptics; #: Score on 4-item geriatric depression scale ≥1; §: mean value of left and right side and based on graduated diapason placed on the tibial tuberosity; **: binocular vision acuity at distance of 5 m with a standard Monoyer letter chart; ††: mean value of the highest value of maximal isometric voluntary contraction strength measured with computerized dynamometers expressed in Newton per square meter; ‡‡: measured at steady state walking with GAITRite system; ¶¶: stride-to-stride variability of stride time measured at steady state walking with GAITRite system; ##: calculated from the formula (Timed up & Go realized – Timed up & Go imagined/((Timed up & Go realized – Timed up & Go imagined)/2) ×100; P-value significant (i.e., < 0.05) indicated in bold.

**Figure 2:**
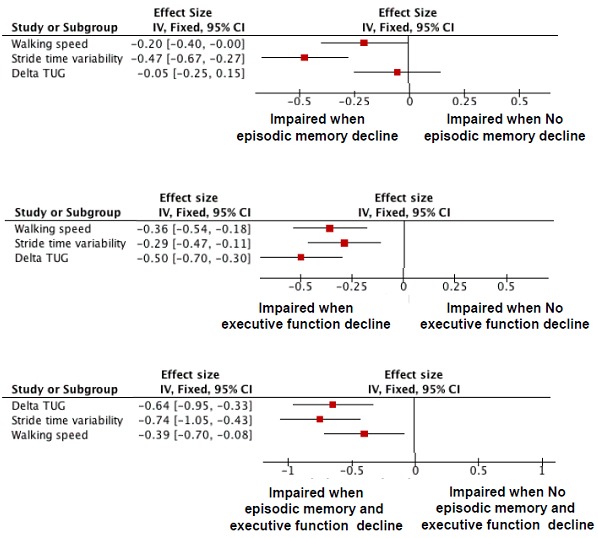
Effect size of the association of gait speed, stride time variability, and delta time of Timed up & Go with lower cognitive performance in memory and executive domains (n = 934). TUG: Timed up & Go; delta time of Timed up & Go calculated from the formula: (Timed up & Go realized – Timed up & Go imagined/((Timed up & Go realized – Timed up & Go imagined)/2) ×100.

Table 2 presents the logistic regressions investigating the association between a low cognitive performance (i.e., episodic memory, executive function, and combination of these both cognitive functions) and each gait parameter (i.e., gait speed, STV, and TUG delta time) adjusted on participants' characteristics. An increase in STV was associated with a lower episodic memory performance (P = 0.001); whereas, an increase in TUG delta was associated with a lower executive performance (P<0.001). A lower gait speed and a higher STV were shown in participants with lower episodic memory and lower executive performance (P = 0.038 and P = 0.019). As shown in Figure 2, the highest effect size was reported for STV among participants with a lower episodic memory performance (effect size  =  −0.47 [95% confidence interval (CI): −0.67;−0.27])and among participants with a combination of lower episodic memory and lower executive performance (effect size  =  −0.74 [95% CI: −1.05;−0.43]); whereas, the highest effect size was reported for TUG delta time among participants with lower executive performance compared to the other participants (effect size  =  −0.50 [95% CI: −0.70;−0.30]).

**Table 2:** Multiple logistic regression analysis showing the association between decline in cognitive performance (dependent variable) and gait performance (independent variable) adjusted for participants' baseline characteristics (n = 934).

	Decline in cognitive performance										
	Episodic memory*				Executive function†				Episodic memory & executive function‡		
	OR	[95%,CI]	P-value		OR	[95%,CI]	P-value		OR	[95%,CI]	P-value
Decrease in walking speed ¶ (cm/s)	1.007	[0.997;1.017]	0.157		1.008	[0.999;1.018]	0.091		1.017	[1.001;1.034]	**0.038**
Increase in stride time variability # (%)	1.153	[1.057;1.257]	**0.001**		1.086	[0.998;1.182]	0.057		1.173	[1.027;1.340]	**0.019**
Increase in delta time of Timed up & Go § (%)	1.002	[0.995;1.008]	0.648		1.015	[1.009;1.022]	**<0.001**		1.010	[1.000;1.020]	0.051

OR: odds ratio; CI: confidence interval;*: abnormal score on the Short Mini-Mental State Examination (score  =  5); †: abnormal score on the clock drawing test; ‡: participants with abnormal score on the Short Mini-Mental State Examination and the clock-drawing test; ¶: measured at steady state walking with GAITRite system; #: stride-to-stride variability of stride time measured at steady state walking with GAITRite system; §: calculated from the formula (Timed up & Go realized – Timed up & Go imagined/((Timed up & Go realized – Timed up & Go imagined)/2) ×100; P-value significant (i.e., <0.05) indicated in bold; all models are adjusted on participants' baseline characteristics (i.e., age, gender, number of drugs used per day, use of psychoactive drugs, depression symptoms, body mass index, lower-limb proprioception, distance vision score and handgrip strength).

**Figure 3:**
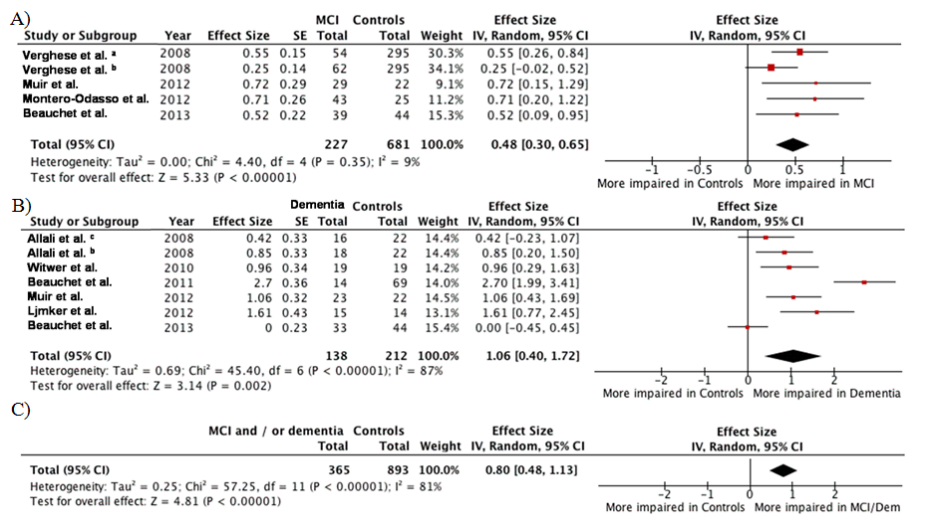
Meta-analyses of studies examining the associations between stride time variability and decline in cognitive performance. Forest plots for effect size of high stride time variability A) in cognitively healthy individual and patients with mild cognitive impairment, B) in cognitively healthy individual and demented patients, C) in cognitively healthy individual and patients with decline in cognitive performance (i.e., mild cognitive impairment or dementia). The square area is proportional to the sample size of each study, and horizontal lines correspond to the 95% confidence interval. The diamond represents the summary value. The vertical line corresponds to 0.0, equivalent to no difference. a: Patients with amnestic Mild Cognitive Impairment. b: Patients with non-amnestic Mild Cognitive Impairment. c: Demented patients with executive dysfunction. d: Demented patients without executive dysfunction.

The meta-analysis was performed on 8 studies with a total of 365 cases (i.e., 227 patients with MCI and 138 patients with dementia) and 893 controls (i.e., CHI) (Figure 3). For patients with MCI, the summary random effect size was 0.48 [95% CI: 0.30;0.65] indicating that STV was overall 0.48 SD higher (i.e. worse) in patients with MCI compared to CHI (Figure 2 A). This represents a moderate association of increased STV with MCI [34]. Using the ‘Common Language Effect Size’ approach of McGraw and Wong, the probability is about 48% that a patient with MCI would have higher gait variability than a CHI if both individuals were chosen at random from a population [27]. For patients with dementia, the summary random effect size was 1.06 [95% CI: 0.40;1.72], underscoring a large association with increased STV. In final, when considering pooled populations with cognitive decline (i.e., patients with MCI or dementia), the summary random effect size was 0.80 [95% CI: 0.48;1.13] highlighting a moderate association in total.

## Discussion

The results of this study of older community-dwellers without a diagnosis of dementia showed that higher STV was associated with a lower cognitive performance in episodic memory and executive function. The results of the meta-analysis confirmed this result by underscoring that higher STV was related to both MCI and dementia. Thus, higher STV appears as the motor phenotype of cognitive decline before and during the course of dementia.

Our findings corroborate that declining gait performances are strongly related to cognitive impairments. Most of the previous studies focused on gait changes in demented patients, and these studies showed that these gait changes are common in the later stages of dementia, with a prevalence reaching 90%, and correspond to various gait changes such as cautious gait or slower gait with a higher fall risk [13], [36]–[41]. Demented older adults exhibit greater gait impairments than those impairments expected as a result of the normal aging process [41], [42]. More recently, it has been reported that gait changes may be detected early in the course of dementia, including at the prodromal stage of MCI, which is a transitional state between normal cognition and dementia [5], [6], [14], [41], [43]. In particular, higher STV has been observed among individuals with MCI [5], [6]. Our results contribute new information by showing that STV is also strongly related to the level of cognitive performance in individuals without dementia. Indeed, the lower cognitive performance they had, the higher STV we observed. Few studies had shown similar results, but the association between specific cognitive domains and gait parameters was divergent. For instance, it has been found that a lower performance on global executive function was associated with slower gait speed [8], [9], [44]. And more recently, it has been reported in older cognitively healthy community-dwellers that higher STV was associated with impairments in information updating and monitoring, which is a subdomain of the executive functions [7]. The fact that we did not find, in the present study, such an association between higher STV and executive dysfunction is probably related to the test used to evaluate executive function. Although less accurate than more comprehensive battery tests, the Clock Drawing Test is more feasible and widely used in clinical practice to address executive functions [45], [46].

Higher STV appeared as a gait change strongly related to decline in cognitive performance in both our original study and the meta-analysis. The reason for the attractiveness of this measure is based on the fact that STV reflects one of the final pathways of the outcomes regulated by the central nervous system. The general assumption is that there is an inverse association between stride time variability and gait stability [1]–[6]. Lower STV reflects automatic processes that require minimal cortical input, and lower STV is associated with efficient and safe gait patterns [6]. Walking is one of the most repetitive and “hard wired” human movements; the normal fluctuations in STV are usually below 3% among healthy adults [1], [2], [6], [9]. Thus, STV appears as a good biomarker of higher levels of gait control and, therefore, highlights the fact that gait should not be considered as a simple automatic motor behavior but a rather complex and higher level of cognitive functioning. Understanding the mechanisms of cognitive decline-related increase in STV seems of particular importance. It is likely that they depend in part on lesions in the basal ganglia, as observed in more severe stages of dementia [36]–[40]. However, since it has been reported that an increase in STV is a surrogate marker of motor power and propulsion abilities among individuals with MCI [1], [2], [5]–[7], it is likely that the involvement of the brain exceeds the subcortical level, and also relates to cortical cognitive dysfunction with subsequent cortical misprocessing of sensorimotor information resulting in higher STV [2], [3], [7]. Because we showed that higher STV was associated specifically with lowest episodic memory performance in our study, we thus suggest that higher STV in non-demented individuals may reflect primarily a dysfunction of cortical sensorimotor control involving the hippocampus. This reasoning is in concordance with the association between higher gait variability and hippocampus dysfunction previously reported [1], [2], [14], [47], [48]. For instance, the approach of gait in terms of brain metabolism by Zimmerman et al. showed that higher stride length variability was associated with lower levels of hippocampal metabolism [14]. Animal models have also shown that hippocampus lesions generated memory disorders as well as limb coordination impairments evaluated by STV [49]. In functional MRI studies, a greater extent of hippocampal activation and a trend toward increased entorhinal cortex activation have been found in MCI patients compared to controls while performing an episodic memory task of encoding, whereas AD patients showed a lower degree of activation in these same regions [50]. In a recent fMRI study comparing mental imagery of gait between healthy older and younger adults, the elderly showed a greater activation in the left hippocampus than the young subjects for a task assessing the precise control of gait [51]. Thus, in line with the hypothesis of a compensatory hippocampal activity in cognitively healthy individuals and those with MCI, the association between higher STV and low memory performance could reflect a pathological compensatory mechanism related to hippocampal dysfunction in prodromal AD.

Slower gait speed was only reported among participants with cumulative memory and executive impairments. The result is consistent with previous studies showing that gait speed decreases in AD and follows the severity of the disease [2], [10], [42]. This change in gait speed has been related to a decrease in stride length and an increase in support time [10], [42] and, thus, provides complementary information compared to STV. Indeed, compared to STV, which seems to be strongly related to highest levels of gait control, gait speed is a global biomarker of gait disturbance related to the central but also the peripheral disturbance of neuromuscular system [52]. Indeed, gait speed is a simple, objective, performance-based measure of lower limb neuromuscular function, which not only allows detection of subtle impairments and preclinical diseases, but is also a sensitive marker of functional capacity in older adults [53]–[55].

Our study also showed that TUG delta time was only related to executive dysfunction. Similar results have been previously reported in older adults [15], but also in patients with schizophrenia [16] and multiple sclerosis [56]. Indeed, global cognitive decline has been related to an important time difference between TUG and iTUG. This result underscore that gait should no longer be considered as a simple automatic motor activity independent from cognition. The exact localization of cortical gait control disorders remains uncertain. Recently, Wang et al. reported using fMRI that gait-activated motor-related areas of the brain, included the supplementary motor area, bilateral precentral gyrus, left dorsal premotor cortex, and cingulated motor area [57]. Different brain areas such as the prefrontal cortex, and in particular the Brodmann area 6 and the posterior supplementary motor cortex, seem to be predominantly implicated in Motor Imagery [58], [59]. One can postulate that our participant presenting the lowest cognitive performance might also have deficits in those regions, which may disturb their motor imagery ability. The latter point could explain the positive association between lowest cognitive performance and delta time reported in our study.

Some limitations need to be noted in our original study. First, the cross-sectional design may be problematic when exploring an association between gait and cognitive performance compared to a prospective cohort study design. The causality and direction in the association of changes in gait with cognitive decline should be carefully interpreted. Thus, our findings should be replicated in a longitudinal cohort study with MCI and/or dementia occurrence information collected prospectively. Second, an abnormal SMMSE score as well as an abnormal clock drawing test could be not sufficient to diagnose satisfactorily memory and executive declines. These both tests are usually used as screening tests rather than diagnostic tests in a general population. A diagnosis of cognitive decline in these two sub-domains usually requires a multidisciplinary meeting involving geriatricians, neurologists and neuropsychologists during which the results of neuropsychological assessment, medical examination, blood tests and brain imaging are discussed. Third, it is possible that some participants recruited in this study were MCI and/or mildly demented participants. Indeed, prevalence estimates of MCI have ranged between 10.7% and 14.5% among samples in previous European studies [60]–[62]. The prevalence estimate in the current study is, at most, three times larger as the previous reports and may represent a methodological difference between studies in identifying people with dementia. The evaluation process in the current study also did not specifically seek to identify individuals who would meet the diagnostic criteria for MCI. Fourth, although we were able to control for many characteristics likely to modify the association between gait and cognitive performance, residual potential confounders might still be present in our study. Fifth, potential limitations of our meta-analysis should be also considered. It was performed on a relatively small number of studies (n = 8), which underscores i) that research on cognitive-related changes in STV is still limited at this time, and ii) a potential publication bias. In addition, while a meta-analysis of effect sizes is equivalent to a meta-analysis of odds ratios -albeit with loss of power- when there is an underlying normal distribution and common variance [63], this assumption may be not entirely correct in the populations selected in our review due to the relatively small sample sizes. Finally, the determined summary effect size should be interpreted with caution due to the substantial heterogeneity, at least for the analysis of patients with dementia. However, the use of a random-effects meta-analysis model controlled this limitation and compensated for the different effect distributions across the different studies [64].

In contrast, our study has a number of strengths. First, it is the largest population based study in older adults that examined the association of gait performance with cognitive performance. Second, compared to previous published studies, the major potential confounders (i.e., age, gender, number of drugs used per day, use of psychoactive drugs, depression symptoms, body mass index, lower-limb proprioception, distance vision score and handgrip strength) in our study were taken into account. Third, all participants had a comprehensive clinical examination and specific gait assessment with the GAITRite system, which is a validated portable gait analysis system that allows simple objective gait measurements. The results of this study should be generalizable to community-dwelling older adults, which improves the knowledge translation potential of the study results.

Our results underscore a specific association between increased STV and cognitive decline from non-demented to demented patients. This result leads to new perspectives in the diagnosis of early stages of dementia such as MCI. Indeed, the diagnosis of MCI is based on a comprehensive neuropsychological assessment exploring various cognitive domains including episodic memory and executive function, but also on blood tests and brain imaging [20]. This diagnosis process is time-consuming and expensive. In addition, there are no gold standards to conclude that an individual has MCI. As interventions appear to be more effective in the early stages of the disease, improving the early diagnosis of dementia at the prodromal stage of MCI is challenging for clinicians. Recently, the use of biomarkers has been proposed to facilitate the early diagnosis of dementia [65]. Biomarkers are defined as indicators of a disease process, and their complementary use to classical neuropsychological tools is essential to this aim [65]. For example, specific proteins in the cerebrospinal fluid (CSF) (e.g., protein tau) constitute validated biomarkers for AD [65]. However, the main limitation of CSF biomarkers is the compulsory invasive examination (i.e., CSF tapping). Compared to these biomarkers, spatio-temporal gait parameters reflecting motor disorders of early-stage dementia could represent non-invasive easily accessible biomarkers to improve the prediction of dementia, and especially in AD [1], [2].

In conclusion, higher STV was the gait parameter with the highest magnitude of association with cognitive decline in both individuals with and without dementia. This finding could be applied as potential biomarker of cognitive decline, which will be useful for the early diagnosis of dementia.

## Supporting Information

Checklist S1 **PRISMA checklist.**
(DOC)

## References

[1] Montero-Odasso M, Verghese J, Beauchet O, Hausdorff JM (2012) Gait and cognition: a complementary approach to understanding brain function and the risk of falling. J Am Geriatr Soc. 60: 2127–36. 23110433 10.1111/j.1532-5415.2012.04209.x PMC3498517

[2] Beauchet O, Allali G, Berrut G, Hommet C, Dubost V, et al (2008) Gait analysis in demented subjects: Interests and perspectives. Neuropsychiatr Dis Treat 4: 155–160. 18728766 10.2147/ndt.s2070 PMC2515920

[3] Allan LM, Ballard CG, Burn DJ, Kenny RA (2005) Prevalence and severity of gait disorders in Alzheimer's and non-Alzheimer's dementias. J Am Geriatr Soc. 53: 1681–1687. 16181166 10.1111/j.1532-5415.2005.53552.x

[4] Verghese J, Wang C, Lipton RB, Holtzer R (2013) Motoric cognitive risk syndrome and the risk of dementia. J Gerontol A Biol Sci Med Sci. 68: 412–418. 22987797 10.1093/gerona/gls191 PMC3593614

[5] Beauchet O, Allali G, Launay C, Herrmann FR, Annweiler C (2013) Gait variability at fast-pace walking speed: a biomarker of mild cognitive impairment? J Nutr Health Aging 17: 235–239. 23459976 10.1007/s12603-012-0394-4 PMC12878871

[6] Beauchet O, Allali G, Thiery S, Gautier J, Fantino B, et al (2011) Association between high variability of gait speed and mild cognitive impairment: a cross-sectional pilot study. J Am Geriatr Soc. 59: 1973–1974. 22091517 10.1111/j.1532-5415.2011.03610_9.x

[7] Beauchet O, Annweiler C, Montero-Odasso M, Fantino B, Herrmann FR, et al (2012) Gait control: a specific subdomain of executive function? J Neuroeng Rehabil 9: 12. 22321772 10.1186/1743-0003-9-12 PMC3308913

[8] Watson NL, Rosano C, Boudreau RM, Simonsick EM, Ferrucci L, et al (2010) Executive function, memory, and gait speed decline in well-functioning older adults. J Gerontol A Biol Sci Med Sci. 65: 1093–1100. 20581339 10.1093/gerona/glq111 PMC2949334

[9] Hausdorff JM, Yogev G, Springer S, Simon ES, Giladi N (2005) Walking is more like catching than tapping: gait in the elderly as a complex cognitive task. Exp Brain Res. 164: 541–548. 15864565 10.1007/s00221-005-2280-3

[10] van Iersel MB, Hoefsloot W, Munneke M, Bloem BR, Olde Rikkert MG (2004) Systematic review of quantitative clinical gait analysis in patients with dementia. Z Gerontol Geriatr 37: 27–32. 14991293 10.1007/s00391-004-0176-7

[11] Kearney FC, Harwood RH, Gladman JR, Lincoln N, Masud T (2013) Dement Geriatr Cogn Disord. 36: 20–35. 10.1159/000350031 23712088

[12] Beauchet O, Allali G, Annweiler C (2009) Gait variability among healthy adults: low and high stride-to-stride variability are both a reflection of gait stability. Gerontology 55: 702–706. 19713694 10.1159/000235905

[13] Sheridan PL, Solomont J, Kowall N, Hausdorff JM (2003) Influence of executive function on locomotor function: divided attention increases gait variability in Alzheimer's disease. J Am Geriatr Soc. 51: 1633–7. 14687395 10.1046/j.1532-5415.2003.51516.x

[14] Zimmerman ME, Lipton RB, Pan JW, Hetherington HP, Verghese J (2009) MRI- and MRS-derived hippocampal correlates of quantitative locomotor function in older adults. Brain Res. 1291: 73–81. 19631621 10.1016/j.brainres.2009.07.043 PMC2747520

[15] Beauchet O, Annweiler C, Assal F, Bridenbaugh S, Herrmann FR, et al (2010) Imagined Timed Up & Go test: a new tool to assess higher-level gait and balance disorders in older adults? J Neurol Sci. 294: 102–106. 20444477 10.1016/j.jns.2010.03.021

[16] Lallart E, Jouvent R, Herrmann FR, Beauchet O, Allali G (2012) Gait and motor imagery of gait in early schizophrenia. Psychiatry Res. 198: 366–370. 22445069 10.1016/j.psychres.2011.12.013

[17] Bridenbaugh SA, Beauchet O, Annweiler C, Allali G, Herrmann F, et al (2013) Association between dual task-related decrease in walking speed and real versus imagined Timed Up and Go test performance. Aging Clin Exp Res. 25: 283–289. 23740587 10.1007/s40520-013-0046-5

[18] Podsiadlo D, Richardson S (1991) The timed “Up & Go”: a test of basic functional mobility for frail elderly persons. J Am Geriatr Soc 39: 142–148. 1991946 10.1111/j.1532-5415.1991.tb01616.x

[19] Annweiler C, Beauchet O, Bartha R, Wells JL, Borrie MJ, et al (2013) Motor cortex and gait in mild cognitive impairment: a magnetic resonance spectroscopy and volumetric imaging study. Brain 136: 859–871. 23436505 10.1093/brain/aws373

[20] Haubois G, de Decker L, Annweiler C, Launay C, Allali G, et al (2013) Derivation and validation of a Short Form of the Mini-Mental State Examination for the screening of dementia in older adults with a memory complaint. Eur J Neurol 20: 588–590. 22913655 10.1111/j.1468-1331.2012.03830.x

[21] Beauchet O, Annweiler C, Verghese J, Fantino B, Herrmann FR, et al (2011) Biology of gait control: vitamin D involvement. Neurology 76: 1617–1722. 21471466 10.1212/WNL.0b013e318219fb08 PMC3100089

[22] Lord SR, Ward JA, Williams P, Anstey KJ (1994) Physiological factors associated with falls in older community-dwelling women. J Am Geriatr Soc. 42: 1110–1117. 7930338 10.1111/j.1532-5415.1994.tb06218.x

[23] Shah A, Herbert R, Lewis S, Mahendran R, Platt J, et al (1997) Screening for depression among acutely ill geriatric inpatients with a short Geriatric Depression Scale. Age Ageing 26: 217–221. 9223718 10.1093/ageing/26.3.217

[24] Sunderland T, Hill JL, Mellow AM, Lawlor BA, Gundersheimer J, et al (1989) Clock drawing in Alzheimer's disease. A novel measure of dementia severity. J Am Geriatr Soc. 37: 725–729. 2754157 10.1111/j.1532-5415.1989.tb02233.x

[25] Kressig RW, Beauchet O (2006) European GAITRite Network Group. Guidelines for clinical applications of spatio-temporal gait analysis in older adults. Aging Clin Exp Res. 18: 174–176. 16702791 10.1007/BF03327437

[26] von Elm E, Altman DG, Egger M, Pocock SJ, Gotzsche PC, et al (2008) The Strengthening the Reporting of Observational Studies in Epidemiology (STROBE) statement: guidelines for reporting observational studies. J Clin Epidemiol 61: 344–349. 18313558 10.1016/j.jclinepi.2007.11.008

[27] Schulz KF, Altman DG, Moher D (2010) CONSORT 2010 statement: updated guidelines for reporting parallel group randomised trials. PLoS Med 7: e1000251. 20352064 10.1371/journal.pmed.1000251 PMC2844794

[28] Montero-Odasso M, Muir SW, Speechley M (2012) Dual-task complexity affects gait in people with mild cognitive impairment: the interplay between gait variability, dual tasking, and risk of falls. Arch Phys Med Rehabil 93: 293–299. 22289240 10.1016/j.apmr.2011.08.026

[29] Ijmker T, Lamoth CJ (2012) Gait and cognition: the relationship between gait stability and variability with executive function in persons with and without dementia. Gait Posture 35: 126–130. 21964053 10.1016/j.gaitpost.2011.08.022

[30] Muir SW, Speechley M, Wells J, Borrie M, Gopaul K, et al (2012) Gait assessment in mild cognitive impairment and Alzheimer's disease: the effect of dual-task challenges across the cognitive spectrum. Gait Posture 35: 96–100. 21940172 10.1016/j.gaitpost.2011.08.014

[31] Wittwer JE, Webster KE, Menz HB (2010) A longitudinal study of measures of walking in people with Alzheimer's Disease. Gait Posture 32: 113–117. 20447826 10.1016/j.gaitpost.2010.04.001

[32] Allali G, Assal F, Kressig RW, Dubost V, Herrmann FR, et al (2008) Impact of impaired executive function on gait stability. Dement Geriatr Cogn Disord 26: 364–369. 18852489 10.1159/000162358

[33] Verghese J, Robbins M, Holtzer R, Zimmerman M, Wang C, et al (2008) Gait dysfunction in mild cognitive impairment syndromes. J Am Geriatr Soc. 56: 1244–1251. 18482293 10.1111/j.1532-5415.2008.01758.x PMC2574944

[34] Egger M, Davey Smith G, Altman D (2001) Systematic Reviews in Health Care: Meta-Analysis in Context, BMJ Publishing Group, London.

[35] Higgins JP, Thompson TS (2002) Quantifying heterogeneity in a meta-analysis. Stat Med 2002 21: 1539–1558. 12111919 10.1002/sim.1186

[36] Camicioli R, Howieson D, Oken B, Sexton G, Kaye J (1998) Motor slowing precedes cognitive impairment in the oldest old. Neurology 50: 1496–1498. 9596020 10.1212/wnl.50.5.1496

[37] Marquis S, Moore MM, Howieson DB, Sexton G, Payami H, et al (2002) Independent predictors of cognitive decline in healthy elderly persons. Arch Neurol 59: 601–606. 11939895 10.1001/archneur.59.4.601

[38] Scarmeas N, Albert M, Brandt J, Blacker D, Hadjigeorgiou G, et al (2005) Motor signs predict poor outcomes in Alzheimer disease. Neurology 64: 1696–16703. 15911793 10.1212/01.WNL.0000162054.15428.E9 PMC3028937

[39] Waldemar G, Dubois B, Emre M, Georges J, McKeith IG, et al (2007) Recommendations for the diagnosis and management of Alzheimer's disease and other disorders associated with dementia: EFNS guideline. Eur J Neurol. 14: e1–26. 10.1111/j.1468-1331.2006.01605.x 17222085

[40] Waite LM, Grayson DA, Piguet O, Creasey H, Bennett HP, et al (2005) Gait slowing as a predictor of incident dementia: 6-year longitudinal data from the Sydney Older Persons Study. J Neurol Sci. 229–230: 89–93. 10.1016/j.jns.2004.11.009 15760625

[41] Allan LM, Ballard CG, Burn DJ, Kenny RA (2005) Prevalence and severity of gait disorders in Alzheimer's and non-Alzheimer's dementias. J Am Geriatr Soc. 53: 1681–1687. 16181166 10.1111/j.1532-5415.2005.53552.x

[42] Scherder E, Eggermont L, Swaab D, van Heuvelen M, Kamsma Y, et al (2007) Gait in ageing and associated dementias; its relationship with cognition. Neurosci Biobehav Rev. 31: 485–497. 17306372 10.1016/j.neubiorev.2006.11.007

[43] Kluger A, Gianutsos JG, Golomb J, Ferris SH, George AE, et al (1997) Patterns of motor impairement in normal aging, mild cognitive decline, and early Alzheimer's disease. J Gerontol B Psychol Sci Soc Sci. 52: P28–39. 10.1093/geronb/52b.1.p28 9008673

[44] Ble A, Volpato S, Zuliani G, Guralnik JM, Bandinelli S, et al (2005) Executive function correlates with walking speed in older persons: the InCHIANTI study. J Am Geriatr Soc. 53: 410–415. 15743282 10.1111/j.1532-5415.2005.53157.x

[45] Nair AK, Gavett BE, Damman M (2010) Clock drawing test ratings by dementia specialists: interrater reliability and diagnostic accuracy. J Neuropsychiatry Clin Neurosci 22: 85–92. 20160214 10.1176/appi.neuropsych.22.1.85 PMC2938787

[46] Pinto E, Peters R (2009) Literature review of the clock drawing test as a tool for cognitive screening. Dement Geriatr Cogn Disord 27: 201–213. 19225234 10.1159/000203344

[47] Scheltens P, Leys D, Barkhof F, Huglo D, Weinstein HC, et al (1992) Atrophy of medial temporal lobes on MRI in “probable” Alzheimer's disease and normal ageing: diagnostic value and neuropsychological correlates. J Neurol Neurosurg Psychiatry 55: 967–972. 1431963 10.1136/jnnp.55.10.967 PMC1015202

[48] Rosano C, Brach J, Studenski S, Longstreth WT, Newman AB (2007) Gait variability is associated with subclinical brain vascular abnormalities in high-functioning older adults. Neuroepidemiology 29: 193–200. 18043004 10.1159/000111582 PMC2824582

[49] Scherder E, Eggermont L, Swaab D, van Heuvelen M, Kamsma Y, et al (2007) Gait in ageing and associated dementias; its relationship with cognition. Neurosci Biobehav Rev 31: 485–497. 17306372 10.1016/j.neubiorev.2006.11.007

[50] Dickerson BC, Sperling RA (2008) Functional abnormalities of the medial temporal lobe memory system in mild cognitive impairment and Alzheimer's disease: insights from functional MRI studies. Neuropsychologia. 46: 1624–1635. 10.1016/j.neuropsychologia.2007.11.030 PMC2760288 18206188

[51] Allali G, van der Meulen M, Beauchet O, Rieger SW, Vuilleumier P, et al. (2013) The Neural Basis of Age-Related Changes in Motor Imagery of Gait: An fMRI Study J Gerontol A Biol Sci Med Sci. (in press). 10.1093/gerona/glt207 24368777

[52] Annweiler C, Schott AM, Montero-Odasso M, Berrut G, Fantino B, et al (2010) Cross-sectional association between serum vitamin D concentration and walking speed measured at usual and fast pace among older women: the EPIDOS study. J Bone Miner Res. 25: 1858–66. 20205167 10.1002/jbmr.80 PMC5005070

[53] Montero-Odasso M, Schapira M, Soriano ER, Varela M, Kaplan R, et al (2005) Gait velocity as a single predictor of adverse events in healthy seniors aged 75 years and older. J Gerontol A Biol Sci Med Sci 60: 1304–1309. 16282564 10.1093/gerona/60.10.1304

[54] Montero-Odasso M, Schapira M, Varela C, Pitteri C, Soriano ER, et al (2004) Gait velocity in senior people. An easy test for detecting mobility impairment in community elderly. J Nutr Health Aging 8: 340–343. 15359349

[55] Lan TY, Deeg DJ, Guralnik JM, Melzer D (2003) Responsiveness of the index of mobility limitation: comparison with gait speed alone in the longitudinal aging study Amsterdam. J Gerontol A Biol Sci Med Sci. 58: 721–727. 12902530 10.1093/gerona/58.8.m721

[56] Allali G, Laidet M, Assal F, Beauchet O, Chofflon M, et al (2012) Adapted timed up and go: a rapid clinical test to assess gait and cognition in multiple sclerosis. Eur Neurol 67: 116–120. 22236807 10.1159/000334394

[57] Wang C, Wai Y, Kuo B, Yeh YY, Wang J (2008) Cortical control of gait in healthy humans: an fMRI study. J Neural Transm 115: 1149–1158. 18506392 10.1007/s00702-008-0058-z

[58] Guillot A, Collet C, Nguyen VA, Malouin F, Richards C, et al (2008) Functional neuroanatomical networks associated with expertise in motor imagery. Neuroimage 41: 1471–1483. 18479943 10.1016/j.neuroimage.2008.03.042

[59] Bakker M, Verstappen CC, Bloem BR, Toni I (2007) Recent advances in functional neuroimaging of gait. J Neural Transm 114: 1323–1331. 17622483 10.1007/s00702-007-0783-8 PMC2797840

[60] Di Carlo A, Baldereschi M, Amaducci L (2000) Cognitive impairment without dementia in older people: prevalence, vascular risk factors, impact on disability. The Italian Longitudinal Study on Aging. J Am Geriatr Soc. 48: 775–782. 10894316 10.1111/j.1532-5415.2000.tb04752.x

[61] Gavrila D, Antunez C, Tormo M (2009) Prevalence of dementia and cognitve impairment in southeastern Spain: the Ariadna Study. Acta Neurol Scand 120: 300–307. 19832772 10.1111/j.1600-0404.2009.01283.x

[62] Nunes B, Silva R, Cruz V, Roriz J, Pais J, et al (2010) Prevalence and pattern of cognitve impairment in rural and urban populations from Northern Portugal. BMC Neurol 10: 42. 20540726 10.1186/1471-2377-10-42 PMC2905352

[63] Chinn S (2000) A simple method for converting an odds ratio to effect size for use in meta-analysis. Stat Med 19: 3127–3131. 11113947 10.1002/1097-0258(20001130)19:22<3127::aid-sim784>3.0.co;2-m

[64] Riley RD, Higgins JP, Deeks JJ (2011) Interpretation of random effects meta-analyses. BMJ 342, d549. 21310794 10.1136/bmj.d549

[65] Dubois B, Feldman HH, Jacova C, Cummings JL, Dekosky ST, et al (2010) Revising the definition of Alzheimer's disease: a new lexicon. Lancet Neurol 9: 1118–1127. 20934914 10.1016/S1474-4422(10)70223-4

